# *Hermetia illucens* Oil in Calf Milk Replacers Sustains Nutrient Digestibility with Subtle Effects on Concentrate Intake Depending on the Inclusion Level

**DOI:** 10.3390/ani16152363

**Published:** 2026-08-02

**Authors:** Iolanda Altomonte, Federica Salari, Marco Ferrara, Tiziana Vivo, Mina Martini

**Affiliations:** 1 Department of Veterinary Sciences, University of Pisa, Viale delle Piagge 2, 56124 Pisa, Italy; federica.salari@unipi.it (F.S.);; 2 Interdepartmental Research Center, Nutrafood, Nutraceuticals and Food for Health, University of Pisa, 56121 Pisa, Italy

**Keywords:** insect-derived oil, dairy calves, dietary fat, apparent digestibility, fecal nutrient excretion, alternative lipid source

## Abstract

Background: This study evaluates whether different fat formulations of cow’s milk replacers (CMRs) for calves, made by replacing palm and coconut oils with *Hermetia illucens* oil, affect the apparent digestibility of the diet and fecal nutrient excretion at weaning. The effects of these formulations on calf intake, growth, and feed efficiency were also assessed. Methods: Three commercial isoenergetic and isonitrogenous milk replacers, which differed only in the fat source, were tested over ten weeks: CMR C (80/20 palm/coconut oil), CMR T20 (80/20 palm/insect oil), and CMR T40 (60/40 palm/insect oil). Twenty-four calves were randomly assigned to three groups. Feces samples were collected individually at 65–69 days of age. Results: The feed efficiency and the apparent digestion of nutrients did not differ significantly among the groups. However, the T20 group tended to have a lower dry matter intake than the C group and lower average body weight than the T40 group. Conclusions: Black soldier fly oil did not impair apparent nutrient digestibility or feed efficiency at weaning. However, the T20 formulation was associated with reduced solid feed intake and lower average body weight compared with those for T40, indicating that formulation and optimal inclusion level require further investigation.

## Simple Summary

Milk replacers are widely used to feed dairy calves during the pre-weaning period, and the type of fat they contain can influence calf nutrition and development. Coconut oil is commonly included because it provides easily digestible medium-chain fatty acids, but more-sustainable alternatives are being sought. Oil derived from black soldier fly (*Hermetia illucens*) larvae is a promising option for animal nutrition. In this study, vegetal oils were partially replaced with black soldier fly larvae oil in commercial calf milk replacers to evaluate its effects on feed intake, growth, nutrient digestibility, and fecal nutrient excretion. The results showed that replacing part of the vegetable oils with insect oil did not negatively affect the nutrient digestibility or the feed efficiency in the calves compared with control. However, the level of insect oil inclusion influenced feed intake patterns and, at one inclusion level, was associated with a lower average body weight during the experimental period. These findings suggest that black soldier fly larvae oil can be an alternative fat source for calf milk replacers, although its optimal inclusion level requires further investigation.

## 1. Introduction

In modern calf rearing systems, cow milk replacers (CMRs) are widely used as a substitute for whole cow’s milk. The key components of CMRs are protein (typically between 16 and 28% of dry matter (DM) depending on the formulation, mainly of dairy origin); fat, which generally makes up 15–25% of dry matter, with variations based on the climate, breed, and target growth rates [1]; and lactose (typically from 40 to 50%) [2].

Because pre-ruminant calves rely heavily on milk as their primary source of nutrients, CMRs should provide highly digestible nutrients to support optimal growth and development. Fat digestibility is particularly important, as fat represents the main source of dietary energy in CMRs. In addition to increasing the energy density of the diet, dietary fat promotes adipose tissue development, which is especially important during periods of environmental or early-life physiological stress, such as cold exposure. Furthermore, fat also supports thermoregulation and facilitates the absorption of fat-soluble vitamins A, D, E, and K, which are essential for immune function, bone growth, and antioxidant defense in pre-ruminant calves [3].

Fat digestibility is influenced by several factors, including fatty acid chain length, degree of saturation, triglyceride structure, and processing methods such as emulsification [4]. In addition, the digestion dynamics of dietary fat is closely linked to the protein composition of the CMR. In the abomasum, milk proteins coagulate to form a clot, and its physical characteristics (e.g., firmness and consistency) influence the rate of gastric emptying and the subsequent release of nutrients into the small intestine [3]. Consequently, both the fat and protein fractions of CMRs contribute to nutrient digestibility and the efficiency of nutrient utilization in pre-ruminant calves.

In pre-ruminant calves, differences in fat digestion and absorption can influence lipid metabolism [4]; tissue development [5]; and, consequently, growth performance, health status, gastrointestinal development, gut microbiota, and immune function [6,7,8]. This aspect is particularly relevant because the fats used in CMRs often differ substantially from those of bovine milk fat, potentially affecting their digestive and metabolic utilization.

Indeed, the fat fraction of CMRs is typically supplied by blends of vegetable oils and other lipid sources, whose triglyceride structure and FA composition differ from those of bovine milk fat [4]. Compared with bovine milk fat, which contains approximately 71% saturated fatty acids (SFAs) and 3% polyunsaturated fatty acids (PUFAs), conventional CMRs generally have a lower proportion of SFAs (52–60% of total FAs) and a higher proportion of PUFAs (7–13% of total FAs) [4].

Traditionally, animal fats such as tallow and lard have been used as fat sources in CMRs outside the European Union [9]. These fat sources are relatively similar to those of bovine milk fat; these fats are generally well accepted by calves when appropriately emulsified. However, their digestibility may be limited unless they are homogenized and supplemented with suitable emulsifiers, such as lecithin [9].

Within the European Union, and in many other regions of the world, vegetable oils such as palm, coconut, and soybean oil are the predominant lipid sources in CMR formulations [9]. Although these ingredients are widely available and economically competitive, their production has raised important environmental and social concerns. Despite recent efforts to improve the sustainability of vegetable oil production, the cultivation of some crops, particularly oil palm and soybean, continues to be associated with deforestation, biodiversity loss, soil and water degradation, greenhouse gas emissions, and adverse socioeconomic impacts on local communities [10].

Given vegetal oil’s environmental limitations, insects are considered an alternative source of feed for animal nutrition [10,11]. Black soldier fly larvae (BSFL *Hermetia illucens*) and mealworms (*Tenebrio molitor*) are the most widely farmed insect species at an industrial scale.

EU regulation [12,13] generally prohibits the use of animal proteins (except for use in fish meal) in feed for any farmed animal. Currently in the European Union (EU), insect meals have been re-allowed [14,15] for aquaculture, poultry, and pigs, whereas insect meals are still banned in ruminant nutrition [13], with only insect oils being allowed [15].

Oils obtained from insect biomass have emerged as promising alternative lipid sources for livestock nutrition [16], offering the opportunity to diversify the fat ingredients currently used in ruminant feeding. However, evidence supporting their use in young ruminants remains scarce.

To date, most studies have evaluated the digestibility of full-fat insect meals or insect-derived oils either in vitro [17] or in adult ruminants [18], whose digestive physiology differs from that of pre-weaned calves. Consequently, the digestibility and metabolic utilization of insect-derived lipids during the pre-weaning period remain poorly understood, despite dietary fat being the primary source of energy and playing a crucial role in growth and gastrointestinal development.

To date, only one study has investigated the inclusion of insect oil in calf milk replacers, and it focused exclusively on growth performance [19]. Therefore, information on the effects of insect oil-based milk replacers on nutrient digestibility and fecal nutrient excretion in pre-weaned calves is still lacking.

The fat content of insects generally ranges from approximately 28% to 40% of dry matter, depending on the species, larval diet, and analytical method used [11,17]. Among insect species, *Hermetia illucens* fat is characterized by a fatty acid profile that shares some similarities with palm oil, particularly the presence of medium-chain SFAs. However, compared with palm oil, H. illucens fat contains a markedly higher proportion of lauric acid (C12:0) and a lower proportion of palmitic acid (C16:0) [11].

Because dietary fatty acid (FA) composition can influence the FA profile of body tissues and potentially affect their physiological functions [20], understanding the digestibility of insect-derived lipids is essential for evaluating their suitability as alternative fat sources in calf nutrition.

Therefore, the aim of the present study was to evaluate whether replacing conventional fat sources (palm and coconut oils) with *Hermetia illucens* oil in calf milk replacers (CMRs) affects the apparent total-tract digestibility of the diet and fecal nutrient excretion at weaning. In addition, the effects of the different milk replacer formulations on feed intake, growth performance, and feed efficiency were assessed.

## 2. Materials and Methods

### 2.1. Animals, Samples, and Diets

The experiment was carried out on a commercial dairy farm in central Italy between September 2023 and January 2024. Animal care and procedures were carried out in accordance with the Guide for the Care and Use of Laboratory Animals and Directive 2010/63/EU for animal experiments, using noninvasive medical procedures. The study was performed with the consent of the animals’ owner.

A total of 24 newborn Holstein heifer calves (body weight 36 ± 3 kg) were included in the trial. At birth, calves were separated from the cow and moved to individual hutches (1.8 × 2.6 m) bedded with straw. The calves were fed 4 L of maternal colostrum within 4 h of birth. A second 2 L feeding was administered at 6 to 12 h after birth, as part of the farm’s routine management procedures. Bedding was changed as needed to maintain calf comfort. All veterinary procedures for the calves were carried out in accordance with the farm’s veterinary health protocol. All calves remained in good health throughout the study. Fecal consistency was assessed using a 4-point scoring system (0 = normal, 1 = soft, 2 = runny, and 3 = watery) [21], and fecal scores remained close to 0 throughout the experimental period.

Ambient temperature ranged from a maximum of 25 °C at the beginning of the trial to a minimum of 6 °C at the end of the trial.

The calves were randomly assigned to 3 treatments (n = 8 heifers per group) for 10 weeks using three commercial isoenergetic and isonitrogenous CMRs (Table S1), which differed only in the fat source. The CMR control (C)included an 80/20 ratio of palm/coconut oil; CMR T20 included an 80/20 ratio of palm/insect oil from the black soldier fly; and CMR T40 included a 60/40 ratio of palm/insect oil from the black soldier fly.

Single batches of experimental CMR were manufactured by a commercial producer using the same emulsifiers, stabilizers, ingredients (except oil sources) and processing conditions.

Calves were fed twice a day (09:00 and 17:00) with CMR powder (15% dilution): 900 g/d from day 1 up to day 56, then 600 g/d up to day 63, and 300 g/d in one single meal at 09:00 up to day 70. In addition, calves were given solid feed, which consisted of pelleted starter feed and chopped straw. The chemical composition and ingredients of the starter feed are reported in Table S2. Solid feed and water were offered ad libitum from day one of the study.

The amount of CMR and the feed offered and refused by the calves were weighed by a scale, and data were recorded every morning before the new feed distribution to determine the dry matter intake (DMI). No spontaneous refusals of liquid CMR occurred during the trial.

Samples of diets and orts were collected at the beginning, middle and end of the trial and stored at −20 °C prior to analysis for proximate nutrients; they were analyzed in duplicate.

Body weight was measured once a week on the same day approximately 3 h after milk feeding using digital scales. Weaning was gradual and induced at about week 10. At the ages of 65, 66, 67, 68, and 69 days, feces were individually collected from each animal 8 h after the feed delivery as reported in the protocol proposed by Cavallini et al. [22] and stored at −20 °C prior to analysis for chemical composition.

### 2.2. Chemical Analyses of Feed, Milk Replacers and Feces

Starter feed, straw, CMRs and individual samples of feces were analyzed in terms of chemical composition, according to the methods of the Association of Official Analytical Chemists (AOAC) to determine DM (method No. 930.15), ash (method No. 942.05) and crude protein (method No. 984.13) [23]. Ether extract content was determined by Soxhlet extraction (method No. 991.36). Neutral detergent fiber (aNDF) was determined in starter feed, straw and individual samples of feces according to EN ISO 16472 [24], by adding α-amylase (Merck, Darmstadt, Germany) and correcting for residual ash content.

A total starch assay kit (Megazyme Ltd., Bray, Co. Wicklow, Ireland) was used to determine the total starch in starter feed, straw and feces (AOAC Method 996.11, AACC Method 76-13.01) [19].

In the CMRs, fat was extracted by the Rose–Gottlieb method [25]. Fatty acid methyl esters (FAMEs) were prepared according to Christie [26] and analyzed by gas chromatography by a PerkinElmer Clarus 480 equipped with a flame ionization detector and a capillary column (TR-FAME 60 m × 0.25 mm ID; film thickness 0.25 μm, Thermo Fisher Scientific, Waltham, MA, USA). The peak areas of individual FAs were identified using a fatty acid standard injection (Food Industry FAME Mix—Restek Corporation, Bellefonte, PA, USA) and quantified as the percentage of total FAs.

Acid-detergent insoluble ash (AIA) was analyzed in feed and in feces according to Liu [27] and used as an internal marker for predicting fecal output and digestibility [28].

The daily marker intake (AIA intake) was calculated using the following formula:

$$AIA\;daily\;intake=\left(\frac{\left[(AIA\;feed\times Q\;feed)-(AIA\;orts\times Q\;orts\right]}{DMI}\right)$$

where

**✓**  AIA feed is the concentration of marker in feed offered (g/kg DM);
**✓**  Q feed is the amount of feed offered daily (kg/day);
**✓**  AIA orts is the concentration of marker in orts (g/kg DM);
**✓**  Q orts is the amount of orts refused daily (kg/day);
**✓**  DMI is the total daily dry matter intake (kg of DM).

The total-tract apparent digestibility (TTAD) of protein, fat, starch, aNDF and minerals was calculated as follows:

$$Protein\;TTAD\left(\%\right)=100-100\times \frac{\mathrm{AIA}\;\mathrm{diet}}{\mathrm{AIA}\;\mathrm{feces}}\times \frac{\mathrm{Fecal}\;\mathrm{protein}}{\mathrm{Feed}\;\mathrm{protein}}$$

$$Fat\;TTAD\left(\%\right)=100-100\times \frac{\mathrm{AIA}\;\mathrm{diet}}{\mathrm{AIA}\mathrm{feces}\;}\times \frac{\mathrm{Fecal}\;\mathrm{fat}}{\mathrm{Feed}\;\mathrm{fat}}$$

$$Starch\;TTAD\left(\%\right)=100-100\times \frac{\mathrm{AIA}\;\mathrm{diet}}{\mathrm{AIA}\;\mathrm{feces}\;}\times \frac{\mathrm{Fecal}\;\mathrm{starch}}{\mathrm{Feed}\;\mathrm{starch}}$$

$$aNDF\;TTAD\left(\%\right)=100-100\times \frac{\mathrm{AIA}\;\mathrm{diet}}{\mathrm{AIA}\;\mathrm{feces}\;}\times \frac{\mathrm{Fecal}\;aNDF}{\mathrm{Feed}\;aNDF}$$

$$Mineral\;TTAD\left(\%\right)=100-100\times \frac{\mathrm{AIA}\;\mathrm{diet}}{\mathrm{AIA}\;\mathrm{feces}}\times \frac{\mathrm{Fecal}\;minerals}{\mathrm{Feed}\;minerals}$$

Daily fecal output (FO) (kg DM/day) was calculated as follows and subsequently used to estimate the daily excretion of nutrients and minerals:

$$FO=\left(\frac{AIA\;daily\;intake}{AIA\;faces}\right)$$

where

**✓**  AIA daily intake is in g/day;
**✓**  AIA feces is the amount of marker in feces (g/kg DM).

### 2.3. Statistical Analysis

Means and standard deviations were calculated for the chemical composition of the starter feed, straw and CMRs.

Data on fecal excretion, TTAD, dry matter intake (DMI), and growth performance were analyzed using a linear mixed model fitted by restricted maximum likelihood (REML) in JMP version 17.7. (SAS Institute Inc., Cary, NC, USA) [29].

The model included treatment (C, T20, and T40), time, baseline body weight, and the treatment × time interaction as fixed effects, with calf included as a random effect. Normality was assessed using the Shapiro–Wilk test on both the raw data and the model residuals. Post hoc pairwise comparisons among least-squares means were performed using Tukey’s Honest Significant Difference (HSD) test.

Statistical significance was declared at *p* < 0.05.

Only the fixed effect of treatment was considered for the analysis of initial and final BW. A *p* value ≤ 0.05 was considered statistically significant.

## 3. Results

Descriptive analysis of the fatty acid profile of the CMRs (Table 1) revealed a numerically lower unsaturated/saturated (UNS/SFA) ratio in the C and T40 groups (0.65 and 0.62) than that in T20 (0.73).

The C and T40 groups showed the highest numerical SFA contents (60.51 and 61.74 g/100 g total FAs, respectively). In contrast, the PUFA content was similar in T20 and T40 (9.50 and 9.90 g/100 g total FAs, respectively) and numerically higher than in the C group (7.19 g/100 g total FAs). Likewise, the MUFA content was similar in the C and T20 groups (32.30 and 32.76 g/100 g total FAs, respectively), whereas it was numerically lower in T40 (28.36 g/100 g total FAs), reflecting the partial replacement of palm oil with insect oil.

Regarding individual fatty acids, the T20 and T40 groups contained lower numerical amounts of caprylic (C8:0) and capric (C10:0) acids than the C group. The T40 formulation showed the highest numerical lauric acid (C12:0) content and the lowest palmitic acid (C16:0) content among the three CMRs, whereas T20 had the lowest numerical myristic acid (C14:0) content. The concentrations of palmitoleic acid (C16:1) and linoleic acid (C18:2 cis9, 12) increased numerically from C to T20 and T40. Conversely, oleic acid (C18:1 cis9) was numerically lower in T40 than in the other CMRs, whereas conjugated linoleic acid (CLA; C18:2 cis9, trans11) was numerically higher in the insect oil-containing CMR.

The omega-3 and omega-6 fatty acid contents were numerically highest in T20 and T40. The concentrations of the essential fatty acid precursors linoleic acid (C18:2 cis9, 12 n-6) and α-linolenic acid (C18:3 n-3) increased numerically with increasing inclusion of insect oil in the CMRs.

Table 2 presents the fecal composition, daily fecal excretion, and apparent total-tract digestibility of calves at the end of the weaning period.

No significant differences were observed in daily fecal nutrient excretion among the treatment groups. Likewise, at the end of the trial, the inclusion of insect oil in the CMR formulations did not adversely affect apparent total-tract nutrient digestibility.

Regarding the overall growth performance of calves throughout the trial (Table 3), no significant differences were observed among the treatment groups in final body weight, average daily gain, or feed conversion efficiency (FCE). However, calves in the T20 group showed numerically lower ADG and FCE than those in the other groups, although these differences were not statistically significant. In contrast, the average body weight over the experimental period was significantly lower (*p* ≤ 0.05) in the T20 group than in the T40 group.

In addition, calves in the T20 group had a significantly lower total DMI (*p* ≤ 0.01) than those in the C group, as well as a significantly lower intake of SFAs from the CMR (*p* ≤ 0.05) than calves in the other treatment groups. Conversely, PUFA intake increased significantly (*p* ≤ 0.05) and progressively with increasing inclusion of insect oil in the milk replacer formulations. Significant treatment × week interactions were observed for most of the variables evaluated.

Table S3 summarizes calf performance and feed intake over the 10-week experimental period. In all groups, the lowest ADG values were observed during the second week of the trial. As expected, body weight (BW), total dry matter intake (DMI), and starter feed intake increased throughout the study, reaching their highest values during weeks 9 and 10 in all groups. Intakes of MUFAs and PUFAs peaked between weeks 4 and 8 in all groups and declined thereafter. Similarly, SFA intake reached its highest values during the same period in the C group, whereas peak SFA intake occurred between weeks 5 and 8 in the T20 group and between weeks 3 and 8 in the T40 group.

**Figure 1:**
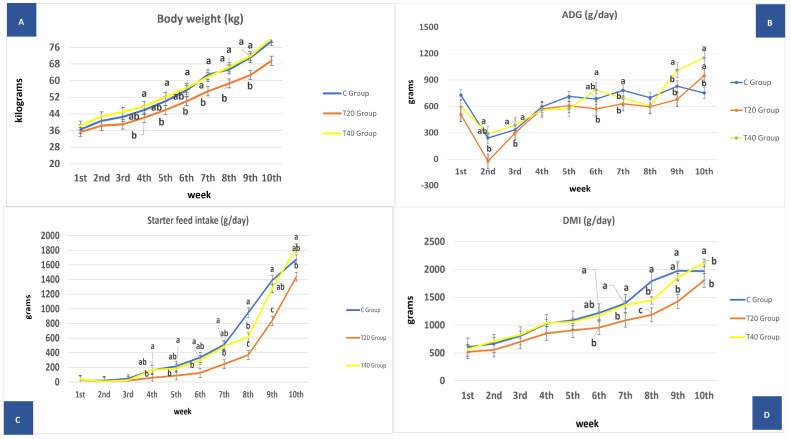
Body weight (**A**), average daily gain (ADG; (**B**)), starter feed intake (**C**), and total dry matter intake (DMI; (**D**)) of calves fed three calf milk replacers (CMRs) throughout the 70-day experimental period. C = control group fed a CMR containing an 80:20 palm oil/coconut oil ratio; T20 = treatment group fed a CMR containing an 80:20 palm oil/insect oil ratio; T40 = treatment group fed a CMR containing a 60:40 palm oil/insect oil ratio. a, b, c = within each week, values with different superscript letters differ significantly (*p* < 0.05).

Body weight (BW; Figure 1A) was significantly lower (*p* ≤ 0.05) in the T20 group than in both the C and T40 groups from weeks 7 to 9. Similarly, average daily gain (ADG; Figure 1B) tended to be lower in the T20 group than in the C group throughout the trial, with significantly lower values (*p* ≤ 0.05) during weeks 1, 2, and 7.

Accordingly, total dry matter intake (DMI; Figure 1D) was significantly lower (*p* ≤ 0.05) in the T20 group than in the C and T40 groups from weeks 7 to 9. Likewise, starter feed intake (Figure 1C) was significantly lower (*p* ≤ 0.05) in the T20 group than in the C group from weeks 5 to 9. In contrast, the DMI pattern observed in the C group was similar to that of the T40 group.

**Figure 2:**
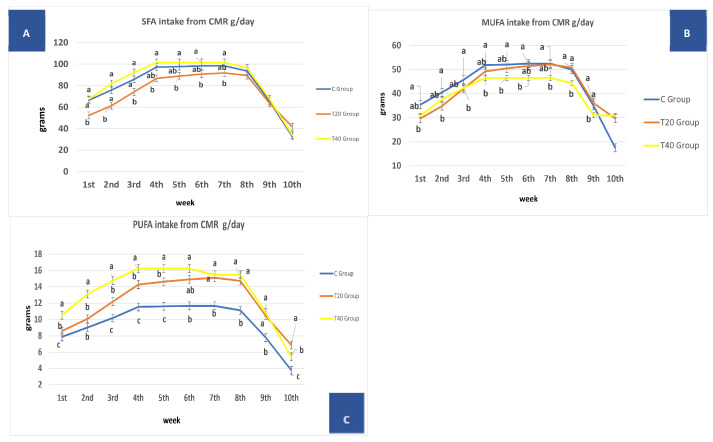
Intake of saturated fatty acids (SFAs; (**A**)), monounsaturated fatty acids (MUFAs; (**B**)), and polyunsaturated fatty acids (PUFAs; (**C**)) from calf milk replacers (CMRs) in calves fed three dietary treatments during the 70-day experimental period. C = control group fed a CMR containing an 80:20 palm oil/coconut oil ratio; T20 = treatment group fed a CMR containing an 80:20 palm oil/insect oil ratio; T40 = treatment group fed a CMR containing a 60:40 palm oil/insect oil ratio. a, b, c = within each week, values with different superscript letters differ significantly (*p* < 0.05).

SFA intake from the CMRs (Figure 2A) was lower (*p* ≤ 0.05) in the T20 group than that in the T40 group up to week 7 and than that in the C group up to week 3. On the other hand, SFA intake did not differ between the T40 and C groups throughout the experimental period. Conversely, PUFA intake (Figure 2C) was higher in the T20 and T40 groups than in the C group throughout the trial (*p* ≤ 0.05).

## 4. Discussion

The fatty acid (FA) composition of the three CMRs reflected the characteristic profiles of palm, coconut, and *Hermetia illucens* oils reported in the literature [30,31]. The higher numerical unsaturated-to-saturated fatty acid (UNS/SFA) ratio and lower proportion of saturated fatty acids (SFAs) observed in the T20 formulation (Table 1) are consistent with the replacement of coconut oil, which is richer in SFAs (approximately 92% of total FAs) [32] than *H. illucens* oil, whose SFA content ranges from 63% to 87%, depending on the larval diet [12]. In contrast, the numerically higher PUFA content of the T20 and T40 formulations reflects the relatively high PUFA concentration of *H. illucens* oil, which can reach up to 35% of total FAs depending on the rearing substrate [11,30]. Conversely, the numerically lower MUFA content of the T40 formulation can be explained by the partial replacement of palm oil, a lipid source naturally rich in MUFAs, with insect oil [30].

The numerically higher proportions of C8:0 and C10:0 observed in the C formulation compared with T20 and T40 reflected the inclusion of coconut oil [31], as these medium-chain fatty acids are absent or present only in negligible amounts in palm oil [30].

The lower numerical C18:1 cis-9 content of the T40 formulation compared with the other CMRs was attributable to the partial replacement of palm oil with *H. illucens* oil. Although oleic acid (C18:1 cis-9) is the predominant MUFA in black soldier fly larval (BSFL) oil [33], its concentration is lower than that reported for palm oil, in which C18:1 cis-9 accounts for approximately 40% of total fatty acids [31]. Furthermore, the detection of conjugated linoleic acid (CLA) in the T20 and T40 formulations is consistent with previous reports describing the presence of CLA in BSFL oil [33].

The numerically higher proportions of omega-3 and omega-6 fatty acids observed in the T20 and T40 formulations reflected the inclusion of *H. illucens* oil. This finding is consistent with previous studies reporting that the omega-3 and omega-6 contents of black soldier fly larvae increase when the insects are reared on substrates naturally enriched in these fatty acids [34]. Because the fatty acid composition of dietary lipids can influence ruminal fermentation and nutrient utilization, modifications in the FA profile of CMRs may also affect nutrient digestibility. Therefore, evaluating nutrient digestibility is essential to determine whether the inclusion of insect oil, despite altering the FA composition of CMRs, influences nutrient utilization in pre-weaned calves. Such information is important for assessing the nutritional suitability of insect oil as an alternative lipid source, with potential implications for improving feed efficiency and potentially reducing nutrient excretion in the environment [35].

The apparent digestibility of crude protein, fiber, and starch observed in the present study (Table 2) was consistent with values previously reported for pre-weaned calves [36]. To date, only a limited number of studies have investigated the digestibility of insect-derived lipids in ruminants, and most have been conducted in vitro. Jayanegara et al. [37] have reported that the inclusion of insect oil reduced substrate digestibility under in vitro conditions. In contrast, no differences in apparent nutrient digestibility were observed among the dietary treatments in the present study, indicating that replacing conventional fat sources with *H. illucens* oil did not impair nutrient utilization in pre-weaned calves. These findings are in agreement with a recent meta-analysis [38], which concluded that the effects of oil supplementation on feed intake, growth performance, and nutrient digestibility in cattle depend on several factors, including the animal category, the type of oil, and the level of dietary inclusion.

Because nutrient digestibility determines the amount of nutrients available for metabolism and tissue accretion, it is a key factor influencing growth performance, particularly average daily gain (ADG) and feed conversion efficiency (FCE). Consistent with the absence of treatment effects on apparent nutrient digestibility, no significant differences in ADG or FCE were observed among the experimental groups. Overall, ADG values recorded throughout the trial (Table 3) were close to the 50th percentile reported for Friesian calves [39], while FCE was comparable to values previously described in Friesian calves fed conventional milk replacers [4].

The lower daily dry matter intake (DMI) observed in the T20 group compared with the other treatments was mainly attributable to a reduced intake of starter feed and hay. Although the lower DMI did not result in significant differences in average daily gain (ADG) or feed conversion efficiency (FCE), it was associated with a lower average body weight (Table 3) than that in the T40 group and with lower body weight values throughout the experimental period (Figure 1A).

The lowest ADG values were observed during the first two weeks of the trial, when CMR represented the major component of the calves’ nutrient intake (Figure 1B). As the milk allowance was progressively reduced from week 8 onwards and the intake of solid feed increased, ADG also increased. During this transition period, calves in the C group showed a more gradual increase in ADG than those in the T20 and T40 groups (Figure 1B). The biological mechanisms underlying these differences remain unclear and warrant further investigation.

The lower ADG and BW observed in the T20 group (Figure 1A,B) may be partly explained by the reduced intake of concentrate feed. Differences in the milk replacer formulation, including potential effects on palatability, may have decreased calves’ motivation to consume starter feed. In addition, the T20 group had the lowest intake of SFAs from the CMR (Figure 2A). Although the underlying mechanisms remain unclear, it is possible that the lower SFA intake contributed to the reduced starter feed intake and, consequently, to the lower ADG and BW observed in this group.

However, these hypotheses were not directly investigated in the present study and should therefore be confirmed by future research.

The various studies investigating the effects of milk fatty acid intake in ruminants have focused on increasing the dietary supply of UNS rather than on reducing SFA intake. For example, studies in adult cattle and calves have reported that dietary supplementation with MUFAs and PUFAs can reduce DMI, possibly through an increase in circulating glucagon-like peptide-1 (GLP-1), a gut-derived hormone involved in the regulation of appetite [40]. Although GLP-1 concentrations were not evaluated in the present study, this mechanism could represent one of the several mechanisms for the reduced feed intake observed in the T20 group and deserves further investigation.

In addition, previous studies have shown that formulating CMRs with a fatty acid profile more closely resembling that of bovine milk fat, for example, by increasing the proportion of medium-chain triglycerides rich in C8:0, can improve growth performance and feed efficiency in calves [4,41]. Interestingly, the fatty acid profile of black soldier fly larvae can be modulated through dietary manipulation of the rearing substrate [42]. This flexibility offers the opportunity to tailor the composition of *H. illucens* oil to better meet the nutritional requirements of pre-weaned calves. Consequently, further research should investigate whether optimizing the fatty acid profile of black soldier fly oil can improve the formulation of calf milk replacers and enhance animal performance.

## 5. Conclusions

This study increases the knowledge on the influence of the acid profile and on the effectiveness of using black soldier fly oil in cow’s milk replacers. Black soldier fly oil did not impair apparent nutrient digestibility or feed efficiency at weaning. However, the T20 formulation was associated with reduced solid feed intake and lower average body weight compared with those for T40, indicating that formulation and optimal inclusion level require further investigation.

## Figures and Tables

**Table 1:** Descriptive statistics of the fatty acid profile of calf milk replacers (CMRs) used in the experiment.

Item	Units	CMR C	CMR T20	CMR T40
		Mean ± SD		
C4:0	g/100 g of total fatty acids	0.11 ± 0.003	0.16 ± 0.02	0.19 ± 0.01
C6:0		0.14 ± 0.001	0.14 ± 0.02	0.18 ± 0.005
C8:0		1.48 ± 0.001	0.40 ± 0.03	0.41 ± 0.01
C10:0		1.57 ± 0.02	0.93 ± 0.12	1.22 ± 0.05
C11:0		0.004 ± 0.002	0.005 ± 0.002	0.006 ± 0.001
C12:0		10.04 ± 0.18	9.19 ± 0.02	16.11 ± 0.77
C13:0		0.02 ± 0.001	0.02 ± 0.004	0.03 ± 0.002
C14:0		5.76 ± 0.07	4.42 ± 0.19	5.88 ± 0.04
C14:1		0.15 ± 0.003	0.18 ± 0.005	0.20 ± 0.007
C15:0		0.19 ± 0.006	0.23 ± 0.006	0.23 ± 0.02
C15:1		0.03 ± 0.004	0.04 ± 0.005	0.04 ± 0.002
C16:0		35.46 ± 0.08	36.55 ± 0.57	32.08 ± 0.17
C16:1		0.22 ± 0.005	0.65 ± 0.007	0.98 ± 0.006
C17:0		0.12 ± 0.0005	0.15 ± 0.003	0.18 ± 0.004
C17:1		0.03 ± 0.003	0.04 ± 0.006	0.05 ± 0.004
C18:0		5.18 ± 0.07	5.12 ± 0.15	4.89 ± 0.25
C18:1 trans-9		0.04 ± 0.0003	0.05 ± 0.01	0.06 ± 0.005
C18:1 trans-11		0.15 ± 0.009	0.19 ± 0.02	0.21 ± 0.03
C18:1 cis-9		31.52 ± 0.10	31.47 ± 0.87	26.67 ± 0.15
C18:2 trans-9, 12		0.01 ± 0.0007	0.04 ± 0.03	0.06 ± 0.007
C18:2 n6		6.61 ± 0.007	8.71 ± 0.26	9.02 ± 0.01
C18:3 n3		0.37 ± 0.002	0.50 ± 0.01	0.57 ± 0.01
C18:3 n6		0.02 ± 0.003	0.04 ± 0.009	0.05 ± 0.001
C20:0		0.27 ± 0.007	0.27 ± 0.001	0.23 ± 0.01
CLA cis-9, trans-11		0.09 ± 0.003	0.14 ± 0.005	0.14 ± 0.01
C20:1		0.14 ± 0.001	0.13 ± 0.003	0.13 ± 0.001
C21:0		0.10 ± 0.000	0.009 ± 0.001	0.0006 ± 0.000
C20:3 n6		0.02 ± 0.003	0.02 ± 0.006	0.01 ± 0.003
C20:3 n3		0.0004 ± 0.000	0.003 ± 0.001	0.002 ± 0.000
C22:0		0.06 ± 0.001	0.07 ± 0.003	0.05 ± 0.008
C22:1		0.004 ± 0.001	0.005 ± 0.001	0.008 ± 0.000
C20:4		0.02 ± 0.002	0.02 ± 0.002	0.02 ± 0.004
C23:0		0.01 ± 0.003	0.01 ± 0.001	0.01 ± 0.004
C22:2		0.002 ± 0.002	0.003 ± 0.000	0.003 ± 0.000
C20:5		0.01 ± 0.001	0.01 ± 0.002	0.004 ± 0.003
C24:0		0.06 ± 0.002	0.05 ± 0.005	0.05 ± 0.003
C24:1		0.005 ± 0.001	0.004 ± 0.000	0.009 ± 0.004
C22:5		0.02 ± 0.002	0.02 ± 0.001	0.01 ± 0.003
C22:6		0.003 ± 0.004	0.004 ± 0.001	0.002 ± 0.001
SFA		60.51 ± 0.11	57.73 ± 1.07	61.74 ± 0.20
MUFA		32.30 ± 0.10	32.76 ± 0.83	28.36 ± 0.18
PUFA		7.19 ± 0.01	9.50 ± 0.24	9.90 ± 0.02
UNS		39.48 ± 0.11	42.27 ± 1.07	38.25 ± 0.20
UNS/SFA		0.65 ± 0.003	0.73 ± 0.03	0.62 ± 0.005
n3		0.40 ± 0.01	0.53 ± 0.01	0.60 ± 0.01
n6		6.68 ± 0.02	8.83 ± 0.22	9.16 ± 0.001
n6/n3		16.70 ± 0.42	16.51 ± 0.12	15.34 ± 0.31

CMR = calf milk replacers; C = calf milk replacers with 80/20 palm/coconut oil ratio; T20 = CMR with 80/20 palm/insect oil ratio; T40 = CMR with 60/40 palm/insect oil ratio; SFAs = saturated fatty acids; MUFAs = monounsaturated saturated fatty acids; PUFAs = polyunsaturated saturated fatty acids.

**Table 2:** Fecal composition, fecal excretion and apparent total-tract digestibility in calves fed 3 milk replacer programs at the end of weaning (65–69 days).

		C Group	T20 Group	T40 Group	RMSE	Effect of Treatment
Item	Units	Least Square Means				*p*-Value
AIA intake daily	g	2.30A	2.13B	2.64A	0.54	0.019
AIA in feces	g/kg DM	19.60	16.91	19.52	5.70	0.631
	Chemical composition of feces					
DM	%	23.97	25.41	25.66	1.62	0.194
Nitrogen	% of DM	2.34	2.34	2.24	0.59	0.812
Protein	% of DM	15.04	15.59	13.87	3.67	0.812
Fat	% of DM	2.03	2.07	2.03	0.41	0.972
aNDF	% of DM	39.95	40.68	41.56	6.23	0.799
Starch	% of DM	0.07	0.20	0.14	0.01	0.087
Ash	% of DM	9.00	9.30	9.32	1.30	0.736
Fecal daily excretions						
Nitrogen excretion	g	17.63	15.69	19.93	10.43	0.711
Protein daily excretion	g	110.22	98.04	124.60	65.20	0.771
Fat	g	18.72	13.07	18.01	9.03	0.193
aNDF	g	333.43	277.28	362.32	149.80	0.465
Starch	g	0.60	1.21	1.27	0.68	0.332
Ash	g	74.47	60.01	80.10	30.52	0.432
Apparent total-tract nutrient digestibility						
Protein	%	71.91	75.78	73.94	13.07	0.779
Fat	%	90.59	92.97	90.91	4.24	0.224
Starch	%	99.84	99.64	99.69	0.12	0.223
aNDF	%	48.42	50.70	47.72	18.41	0.930
Mineral	%	61.27	65.23	59.36	13.45	0.634

CMR = calf milk replacers; C = control group feeding on CMR with 80/20 palm/coconut oil ratio; T20 = treatment group feeding on CMR with 80/20 palm/insect oil ratio; T40 = treatment group feeding on CMR with 60/40 palm/insect oil ratio; RMSE = root mean square error; DM = dry matter; aNDF = amylase-treated neutral detergent fiber.

**Table 3:** Overall performance of calves fed 3 milk replacers (CMRs) over the 70-day trial period.

Item	Units	C Group	T20 Group	T40 Group		Effect of Treatment	Time × Treatment
		Least Square Means			RMSE	*p*-Value	*p*-Value
Total DMI	g DM/day	1256.19A	999.32B	1215.61A	307.80	0.004	≤0.001
Starter feed intake	g DM/day	531.97A	322.16B	491.62AB	306.64	0.004	≤0.001
CMR powder intake	g DM/day	720.49	678.53	726.08	137.93	0.231	≤0.001
SFA intake from CMR	g DM/day	81.10a	74.03b	84.45a	15.37	0.016	0.0001
MUFA intake from CMR	g DM/day	43.27	42.02	38.77	8.66	0.054	0.0001
PUFA intake from CMR	g DM/day	9.46c	12.19b	13.51a	2.29	0.016	0.0001
UNS intake from CMR	g DM/day	52.90	54.20	52.31	10.36	0.685	0.0001
ADG	g/day	647.50	537.94	663.73	285.00	0.124	0.0001
Initial BW	kg	35.58	34.92	37.92	3.22	0.269	-
Final BW	kg	80.74	70.54	82.55	8.57	0.055	-
Average BW	kg	53.47ab	48.39b	54.91a	2.97	0.0359	≤0.001
FCE	-	0.69	0.60	0.68	0.11	0.779	0.779

A,B = within a row, means without a common superscript differ at *p* ≤ 0.01. a, b, c = within a row, means without a common superscript differ at *p* ≤ 0.05. CMR = calf milk replacers; C = control group feeding on CMR with 80/20 palm/coconut oil ratio; T20 = treatment group feeding on CMR with 80/20 palm/insect oil ratio; T40 = treatment group feeding on CMR with 60/40 palm/insect oil ratio; RMSE = root mean square error; DMI = dry matter intake; DM = dry matter; SFAs = saturated fatty acids; MUFAs = monounsaturated saturated fatty acids; PUFAs = polyunsaturated saturated fatty acids; BW = body weight; ADG = average daily weight gain; FCE = feed conversion efficiency.

## Data Availability

The data presented in this study are available from the corresponding author upon reasonable request.

## Supplementary Materials

The following supporting information can be downloaded at https://www.mdpi.com/article/10.3390/ani16152363/s1: Table S1: Chemical composition and energy content of the calf milk replacers (CMRs) used in the experiment. Table S2: Chemical composition of starter feed and straw used in the experiment. Table S3. Weekly performance and intake of calves fed 3 milk replacers (CMRs) over the 70-day trial.
