# Supplementary material for: Hermetia illucens Oil in Calf Milk Replacers Sustains Nutrient Digestibility with Subtle Effects on Concentrate Intake Depending on the Inclusion Level
Source: Animals (Basel). 2026 Aug 2;16(15):2363. doi: 10.3390/ani16152363 (PMC13466035; doi:10.3390/ani16152363)
Supplement: Supplementary file 1 [file animals-16-02363-s001.zip › animals-4431971-supplementary.pdf]

Table S1. Chemical composition and energy content of the calf milk replacers (CMR) used in the experiment

	CMR C	CMR T20	CMR T40
		Means± SD	
ME (MJ/kg) =	18.80	18.97	19.12
DM (%)	96.74±1.14	97.41±0.45	97.57±0.39
CP (% of DM)	25.11±0.41	24.73±0.49	25.08±0.27
Fat (% of DM)	18.6±0.24	18.9±0.64	18.8±0.48
Carbohydrates (% of DM)	45.4	46.1	46.89
Ash (% of DM)	7.59± 0.12	7.68± 0.25	6.80±0.82

CMR C= cow milk replacer with 80/20 palm/coconut oil ratio

CMR T20= cow milk replacer with 80/20 palm/insect oil ratio.

CMR T40= cow milk replacer with 60/40 palm/insect oil ratio

ME (MJ/kg)=(proteinX 16.7) + (fat x 37.7)+ (Carbohydrates X 16.7)/10^6

Carbohydrates were calculated as follow: DM-(fat+crude protein+ash); DM= dry matter; CP= crude protein; NDF=Neutral detergent fiber

Table S2. Chemical composition of starter feed and straw used in the experiment

	**Starter feed**<br>**mean±SD**	**Straw**<br>**mean±SD**
DM (%)	90.08 ±0.78	91.60±0.28
CP (% of DM)	19.82±0.88	9.31±0.80
Fiber (% of DM)	11.01±1.1	39.84±3.74
aNDF (% of DM)	32.73±0.37	70.62±0.61
Starch (% of DM)	21.19±0.19	1.28±0.10
Fat (% of DM)	3.67±0.01	0.70±0.02
Ash (% of DM)	8.35±0.12	15.81±0.12

Ingredients of the starter feed: Wheat middlings, Flaked corn and barley, Sunflower seed meal, Barley, Wheat bran, Soybean meal (dehulled,), Soybean hulls, Beet molasses, Rice husks, Calcium carbonate from ground limestone, Crushed dried carob, Sodium chloride, Corn, Sodium bicarbonate, Inactivated yeasts (Saccharomyces Cerevisiae).

Additives per Kg: Vitamin A 14,280 IU, Vitamin D3 2,000 IU, Vitamin E (all-rac-alpha-tocopheryl acetate) 88 mg, Vitamin B1 1.0 mg, Riboflavin or Vitamin B2 0.50 mg, Vitamin B6 0.50 mg, Vitamin B12 / cyanocobalamin 0.034 mg, Niacin 4.00 mg, Calcium D-pantothenate 1.0 mg, Biotin 0.002 mg, Beta-carotene 0.08 mg Trace elements: Copper(II) sulfate pentahydrate - Cu 17.0 mg, Iron(II) sulfate monohydrate - Fe 27.9 mg, Calcium iodate anhydrous - I 1.2 mg, Manganese(II) oxide - Mn 74.4 mg, Sodium selenite - Se 0.31 mg, Zinc oxide - Zn 39 mg, Selenium yeast Saccharomyces cerevisiae NCYC R397, inactivated - Se 0.20 mg Binders: Sepiolite 490 mg, Sepiolite clay 1,139 mg

DM= dry matter; CP= crude protein; aNDF= amylase-treated neutral detergent fiber

Table S3. Weekly performance and intake of calves fed 3 milk replacer (CMR) over the 70 day trial.

Item	Units	Group	week 1	2	3	4	5	6	7	8	9	10	RMSE	Time
			Least square means											
DMI intake	g of DM /day	C	605.91E	668.01E	804.63ED	1027.69D	1089.75D	1219.55D	1390.02C	1789.73B	1975.94A	1970.64A	307.80	≤0.001
		T20	521.22E	555.96E	700.01ED	851.07D	906.68D	955.07C	1086.54C	1185.21C	1426.43B	1805.06A		
		T40	569.00G	710.00F	825.21E	1037.24D	1057.82D	1170.00D	1363.23C	1444.11C	1858.96B	2120.55A		
Starter feed and hay intake	g of DM /day	C	23.60F	16.62F	47.39F	162.82EF	214.73E	335.34E	509.93D	946.40C	1388.96B	1673.89A	306.64	≤0.001
		T20	23.41D	9.27D	20.48D	57.52D	87.33D	124.47D	245.06CD	368.89C	837.26B	1437.76A		
		T40	33.58E	6.74E	24.00E	164.83E	185.63E	299.77D	489.96C	614.33C	1278.61B	1828.99A		
CMR intake	g of DM / day	C	588.93D	673.80C	762.14B	863.30A	867.11A	873.00A	873.00A	831.00A	582.00D	289.86E	137.93	0.000
		T20	478.94D	562.25C	679.52B	794.01A	814.45A	830.73A	841.12A	819.30A	582.00B	382.96E		
		T40	565.20E	704.29C	793.67B	873.00A	873.00A	873.00A	873.00A	831.00A	582.00D	292.21F		
SFA intake from CMR	g /day	C	66.29D	75.85C	85.79B	97.18A	97.61A	98.31A	98.27A	93.59A	65.51D	32.63E	15.37	≤0.001
		T20	52.25E	61.35D	74.14C	86.63B	88.86A	90.64A	91.77A	89.40A	63.50D	41.78F		
		T40	67.17C	81.76B	92.14A	101.34A	101.35A	101.35A	101.35A	96.52A	67.56C	33.92D		
MUFA intake from CMR	g /day	C	35.37D	40.47C	45.77B	51.85A	52.08A	52.43A	52.43A	49.93AB	34.95D	17.47E	8.66	≤0.001
		T20	29.66D	34.82C	42.09B	49.18A	50.44A	51.45A	52.09A	50.74A	36.04C	29.66D		
		T40	30.86C	37.54B	42.31AB	46.53A	46.53A	46.53A	46.53A	44.31AB	31.02C	30.86D		
PUFA intake	g/ day	C	7.87D	9.01C	10.19B	11.54A	11.60A	11.67A	11.67A	11.12A	7.78D	3.78F	2.29	≤0.001
		T20	8.59C	10.09B	12.20	14.26A	14.62A	14.91A	15.10A	14.71A	10.45B	6.87D		
		T40	10.52C	13.10B	14.77C	16.24A	16.24A	16.24A	15.47A	15.47A	10.83C	5.43D		
UNS intake from CMR	g/ DM per day	C	43.25D	49.48C	55.97B	63.39A	63.67A	64.13A	64.11A	61.05A	42.74D	21.28E	10.36	≤0.001
		T20	38.26D	44.92C	54.29B	63.43A	65.07A	66.37A	67.20A	65.45A	46.50C	30.59E		
		T40	41.61C	50.64B	57.07A	63.78A	62.78A	62.78A	62.78A	59.79A	41.85C	21.01D		

BW	kg	C	35.77I	40.30H	42.08G	44.67F	48.80E	53.25E	58.44D	65.77C	69.70B	76.02A	2.97	≤0.001
		T20	35.36F	38.51G	38.55G	40.66F	44.67E	48.33E	52.84D	57.34C	61.25B	66.44A		
		T40	38.25L	42.08I	44.23H	46.91G	50.83F	54.89E	60.00D	65.23C	69.65B	77.01A		
ADG	g/day	C	726.19A	214.04C	333.52BC	595.36B	711.93A	683.25B	780.40A	498.35B	828.28A	751.38A	285.00	≤0.001
		T20	505.78C	-23.09E	297.57D	571.29C	606.88B	571.40B	629.04B	594.81B	678.59B	947.80A		
		T40	592.33C	285.71E	384.35D	559.51C	571.43C	785.71B	690.48BC	607.14C	1011.90A	1150.57A		

A, B, C, D, E, F, G, H, I, L= Within a row, means without common letters differ at P ≤ 0.01. CMR=cow milk replacer; C=control group feeding CMR with 80/20 palm/coconut oil ratio;T20= treatment group feeding CMR with 80/20 palm/insect oil ratio; T40= treatment group feeding CMR with 60/40 palm/insect oil ratio RMSE=root mean square error;DM=dry matter intake; SFA = saturated fatty acids ; MUFA = monounsaturated saturated fatty acids; PUFA = polyunsaturated saturated fatty acids BW= body weight; ADG= Average daily weight gain; FCE=Feed conversion efficiency.
